# Paradoxical effects of daytime sleepiness and memory in African Americans at risk for Alzheimer’s disease

**DOI:** 10.1093/sleep/zsad301

**Published:** 2023-11-27

**Authors:** Brendan P Lucey

**Affiliations:** Department of Neurology, Washington University School of Medicine, St Louis, MO, USA

Alzheimer’s disease (AD) is a progressive neurodegenerative disease characterized by the deposition of amyloid-β (Aβ) as insoluble extracellular plaques, intracellular aggregates of tau, neuronal loss, and cognitive impairment. Amyloid deposition is a key early step in AD pathogenesis that begins ~15–20 years before the onset of cognitive impairment [1]. Determining if an individual with cognitive impairment has accumulated amyloid pathology (i.e. amyloid-positive) is critical to differentiating AD from other causes of dementia for both diagnosis and treatment. For instance, new anti-amyloid therapies, such as lecanemab [2], reduce amyloid plaques, and slow cognitive decline in individuals with evidence of increased amyloid deposition and cognitive impairment (i.e. symptomatic AD). There are multiple fluid and imaging biomarkers validated to measure amyloid pathology in patients including: cerebrospinal fluid (CSF) Aβ42 concentration [3]; CSF ratios for Aβ42/40, total tau/Aβ42, and phosphorylated tau (p-tau)/Aβ42 [4, 5]; and positron emission tomography (PET) neuroimaging using radiotracers for fibrillar Aβ [6, 7]. Recently, highly sensitive and specific blood-based markers for amyloid pathology have been validated and are increasingly used in clinical practice [8–13].

Substantial evidence supports a bi-directional relationship between sleep and AD: disrupted sleep is associated with increased risk of developing AD and AD pathology is associated with sleep disturbances [14]. For example, short sleep duration [15], decreased sleep efficiency [16], and daytime sleepiness [17] have been associated with amyloid pathology even in cognitively unimpaired older adults although the relationship between sleep duration and amyloid PET imaging was not replicated in one study [18]. Establishing causation and directionality between sleep and AD has been challenging for several reasons including the prolonged asymptomatic period when amyloid pathology accumulates in the absence of cognitive deficits and the lack of longitudinal observational studies of participants well-characterized with standardized cognitive testing, biomarkers for AD pathology, and sleep assessments. Furthermore, multiple factors affect both sleep and AD risk including age, race, and medical comorbidities.

Growing evidence has found that both fluid and imaging biomarkers for amyloid pathology vary by race or ethnicity. For instance, a secondary analysis of >17 000 individuals from the Imaging Dementia-Evidence for Amyloid Scanning study who had mild cognitive impairment or dementia found that Asian, Hispanic, or African American individuals were 53%, 32%, and 29% less likely to have a positive amyloid PET scan than white individuals even after matching for multiple factors such as age, sex, level of education, medical comorbidities, and others [19]. Additional studies have shown the effects of race on AD biomarkers including: CSF tau, CSF p-tau181, plasma p-tau181, plasma p-tau231, and neurofilament light chain [20–22]. Importantly, racial differences in plasma AD biomarkers were found using self-reported race but not genetic ancestry indicating the differences are account for by non-genetic factors [23].

African Americans are at increased risk of AD [24] and poorer sleep health [25] and are under-represented in AD studies, therefore further investigations are urgently needed to determine if the relationship between sleep and AD differs by race.

In Cook et al. [26], 147 cognitively unimpaired self-reported African American participants had cross-sectional measurements of self-reported sleep duration and daytime sleepiness (Epworth Sleepiness Scale), standardized cognitive assessments, and plasma Aβ40, Aβ42, and Aβ42/40 ratio. Fifty-seven participants had longitudinal plasma Aβ measurements and cognitive assessments. After adjusting for age, sex, *APOEε4* allele count, years of education, and body mass index, neither self-reported sleep duration and daytime sleepiness were associated with either cross-sectional or longitudinal plasma Aβ markers. However, self-reported sleep duration was negatively associated with Trail Making Test-B completion time indicating that fewer hours of sleep predicted slower task performance but was not significant longitudinally. In contrast, greater daytime sleepiness was associated with worse overall performance on the Rey Auditory Verbal Learning Test (RAVLT) but there was no association cross-sectionally. Intriguingly, greater daytime sleepiness was paradoxically associated with less decline in recall performance on the RAVLT over time. This paradoxical relationship between daytime sleepiness and episodic memory measured by the RAVLT suggests that declines in cognitive performance due to daytime sleepiness may establish a new lower level of performance but does not otherwise affect the rate of decline and may increase with more restorative sleep.

This study is an important contribution to the field because it is the first study of the relationships between sleep, plasma Aβ, and cognitive performance in African Americans. There are several important limitations, however. A major limitation is the lack of established amyloid biomarkers such as CSF Aβ42/40 ratio or amyloid PET imaging to compare to the findings using plasma Aβ. Plasma assays for Aβ, p-tau, and other AD biomarkers are rapidly evolving and validated for measuring amyloid pathology. Given their recent development, plasma AD biomarkers and the factors that affect their measurement are less well-understood. In addition to the racial differences in plasma AD biomarkers described earlier, chronic kidney disease, myocardial infarction, and stroke are associated with higher plasma p-tau levels [27]. Several studies have also shown varying effects of sleep on plasma AD biomarkers. Sleep loss acutely increased the concentrations of Aβ, tau, and p-tau in CSF but decreased them in blood; the CSF and plasma Aβ42/40 ratios were unaffected [28–30]. Other studies have shown conflicting results with acute sleep loss increasing plasma Aβ40 and decreasing plasma Aβ42/40 ratio [31], or not affecting levels at all [32]. Future studies of large diverse cohorts with paired CSF and plasma samples are needed to establish the relationships between race, sleep, cognitive performance, and other factors to plasma AD biomarkers and how those relationships differ from CSF AD biomarkers.

Another limitation is that only subjective self-reported sleep measures were available for analysis. As the authors noted, these sleep measures were retrospective and susceptible to bias. The authors were unable to replicate a U- or inverse U-shaped relationship between sleep and cognitive performance that has been observed with both subjective and objective sleep measures [33, 34]. For example, short but not long sleep duration was associated with worse Trail Making Test-B performance in this study. Differences between Cook et al. and previous studies include the smaller sample size (especially for the longitudinal measures), a larger number of African American participants, and lack of objective sleep measures. These differences strongly suggest that further investigations are needed. Future studies with a greater number of diverse participants and objectively measured sleep by either actigraphy or polysomnography are needed to test these findings and may impact the use of cognitive performance as an outcome measure in AD trials using a sleep intervention.

Despite these limitations, the study has multiple strengths including a well-characterized cohort of African American participants with a subset having longitudinal assessments. Additional studies in larger cohorts of African American participants, ideally including paired plasma and CSF AD biomarker measurements, are critically needed to confirm these findings and further test for racial differences between sleep, AD biomarkers, and cognitive performance.
